# Comparative analysis and prediction of nucleosome positioning using integrative feature representation and machine learning algorithms

**DOI:** 10.1186/s12859-021-04006-w

**Published:** 2021-06-02

**Authors:** Guo-Sheng Han, Qi Li, Ying Li

**Affiliations:** 1 Department of Mathematics and Computational Science, Xiangtan University, Xiangtan, 411105 Hunan China; 2 Key Laboratory of Intelligent Computing and Information Processing of Ministry of Education and Hunan Key Laboratory for Computation and Simulation in Science and Engineering, Xiangtan University, Xiangtan, 411105 Hunan China

**Keywords:** Nucleosome classification, Frequency chaos game representation, Support vector machine, Extreme learning machine, Extreme gradient boosting, Convolutional neural networks

## Abstract

**Background:**

Nucleosome plays an important role in the process of genome expression, DNA replication, DNA repair and transcription. Therefore, the research of nucleosome positioning has invariably received extensive attention. Considering the diversity of DNA sequence representation methods, we tried to integrate multiple features to analyze its effect in the process of nucleosome positioning analysis. This process can also deepen our understanding of the theoretical analysis of nucleosome positioning.

**Results:**

Here, we not only used frequency chaos game representation (FCGR) to construct DNA sequence features, but also integrated it with other features and adopted the principal component analysis (PCA) algorithm. Simultaneously, support vector machine (SVM), extreme learning machine (ELM), extreme gradient boosting (XGBoost), multilayer perceptron (MLP) and convolutional neural networks (CNN) are used as predictors for nucleosome positioning prediction analysis, respectively. The integrated feature vector prediction quality is significantly superior to a single feature. After using principal component analysis (PCA) to reduce the feature dimension, the prediction quality of H. sapiens dataset has been significantly improved.

**Conclusions:**

Comparative analysis and prediction on H. sapiens, C. elegans, D. melanogaster and S. cerevisiae datasets, demonstrate that the application of FCGR to nucleosome positioning is feasible, and we also found that integrative feature representation would be better.

## Background

The nucleosome is the basic structural unit of eukaryotic chromatin. It is formed by the combination of histones and DNA. The core is an octamer formed by two copies of each histones H2A, H2B, H3 and H4, DNA is wound around it about 1.65 turns. Among them, the DNA wrapped around the octamer is called core DNA, which is 147 base pairs in length; the DNA sequence that connects two adjacent nucleosomes is called linker DNA, which ranges from 20 to 60 base pairs [1]. In eukaryotic cells, nucleosomes play a crucial role in the process of genome expression, DNA replication, DNA repair and transcription [2–6]. In addition, studies have demonstrated that abnormal histone modifications in the nucleosome structure are directly related to diseases such as tumors [7] and lupus erythematosus [8]. Therefore, the mechanism of nucleosome positioning in DNA sequence has an extremely important research value, which is also one of the hot spots in current epigenetics research.

The precise position of the nucleosome on the DNA sequence in the whole genome is called nucleosome positioning. Early experiments mainly used micrococcal nuclease to process chromatin to achieve nucleosome positioning [9]. In recent years, benefiting from the development and application of high-throughput experimental techniques, such as chromatin immunoprecipitation-chip (ChIP-chip), chromatin immunoprecipitation sequencing (ChIP-Seq), many breakthroughs have been made in nucleosome positioning experiments. The nucleosome positioning maps of different species such as Saccharomyces cerevisiae [10, 11], Homo sapiens [12], Caenorhabditis elegans [13], Drosophila melanogaster [14], etc. have been obtained, which provides a large amount of data basis for researchers to carry out theoretical research and prediction.

Much of the research in nucleosome positioning is based on DNA sequence analysis [15, 16]. The DNA sequence consists of four nucleotides: A, T, C and G. Studies have shown that the affinity between genomic DNA sequences and histones is clearly dependent on sequence order, which indicates that the DNA sequence order does affect the position of nucleosome formation. Although some provide the support that nucleosome positioning is affected by multiple factors such as DNA sequence, ATP-dependent nucleosome remodeling enzymes and transcription factors [17, 18]. Many researchers used sequence analysis methods to express nucleosome DNA sequence characteristics and then performed nucleosome positioning and recognition.

In the past decade, with the popularity of machine learning algorithms, a multitude of computational models based on DNA sequence information have been proposed. Chen et al. proposed the "iNuc-Physchem" nucleosome prediction model using 12 physicochemical features of DNA, which identified the core DNA and linker DNA of the yeast genome nucleosome [19]. Later, the research group also established a biophysical model based on the deformation energy of DNA sequences to predict the sequence of nucleosomes [20]. Guo et al. used pseudo k-tuple nucleotide composition to successfully express the feature vector of the DNA sequence, and used the support vector machine (SVM) classifier to train H. sapiens, C. elegans and D. melanogaster [21]. 3LS model used similar methods and combined the distribution of different numbers of nucleotide combinations in the sequence to further improve the prediction accuracy [22]. ZCMM model based on the Z-curve (z-curve) theory and the position weight matrix (PWM), the prediction performance is excellent on D. melanogaster [23].

Deep learning is also applied to nucleosome positioning and achieved good prediction quality. These deep learning models all used one-hot encoding. Gangi et al. [24] constructed a deep learning model that integrates convolutional layers and long short-term memory networks. LeNup model added the Inception module and gated convolutional network to the convolutional neural network to improve the nucleosome positioning [25].

In this work, we firstly will use frequency chaos game representation to construct DNA sequence features. This feature representation method has not been used in nucleosome positioning before. Secondly, we also integrated FCGR with other feature vectors and adopted the principal component analysis (PCA) algorithm to achieve the feature dimensionality reduction. Finally, various machine learning algorithms such as support vector machine (SVM), extreme learning machine (ELM), extreme gradient boosting (XGBoost), multi-layer perceptron (MLP), and convolutional neural networks (CNN) will be used to perform comparative analysis and prediction of nucleosome positioning.

## Results

### Rule of performance evaluation

Cross validation is a statistical analysis method used to validate the model. The basic idea is to divide the original data into a training set and a test set. First, use the training set to train the model, and then use the test set to test the classification or prediction performance of the obtained model. In this work, we used K-fold cross-validation to evaluate the performance of the predictor through four parameters: sensitivity ($S_{n}$), specificity ($S_{p}$), accuracy (ACC), and Mathew's correlation coefficient (MCC). The specific definition are as follows:

1 $$\left\{ {\begin{array}{*{20}c} {S_{n} = \frac{TP}{{TP + FN}}} \\ {S_{p} = \frac{TN}{{TN + FP}}} \\ {ACC = \frac{TP + TN}{{TP + TN + FP + FN}}} \\ {MCC = \frac{TP \times TN - FP \times FN}{{\sqrt {(TP + FN) \times (TP + FP) \times (TN + FN) \times (TN + FP)} }}} \\ \end{array} } \right.$$

where TP, TN, FP and FN are the numbers of true positives, true negatives, false positives and false negatives, respectively [25]. $S_{n}$ is the true positive rate. When $S_{n}$ = 1, it means that all core DNA of nucleosomes have been correctly predicted.${ }S_{p}$ is true negative rate. When $S_{p}$ = 1, it means that all linker DNAs are correctly predicted. ACC reflects the ratio of the number of correctly predicted samples of each category to the total sample. MCC comprehensively evaluates the prediction results. MCC ∈ [− 1,1]. MCC =  − 1 means that the correlation is completely opposite. MCC = 1 means that the prediction result is completely correlated with the true category. MCC = 0 means that the prediction is completely random.

Receiver operating characteristic curve (ROC curve) and area under curve (AUC) are often used to evaluate the pros and cons of a binary classifier. Area under curve (AUC) is the area under the Roc curve, usually between 0.5 and 1. As a value, AUC can be used to evaluate the quality of the classifier more intuitively. The larger the AUC value, the better. Taking into account the length of the paper, this paper only calculates the AUC value and does not draw the ROC curve one by one.

### Performance of predictors

According to the characteristics of FCGR described above, the different values of K nucleotide will affect the feature expression of the DNA sequence [26]. A large K value means a high feature dimension. And generally, high-dimensional features are relatively sparse, and the fitting quality may not be outstanding. Obviously, choosing an appropriate K value will have a greater impact on the classification effect of each classifier. Some studies have combined DNA sequence features [22, 23, 27, 28]. Similarly, FCGR can also use different combinations of K nucleotide values as feature vectors.

### Feasibility of FCGR

**Fig. 1:**
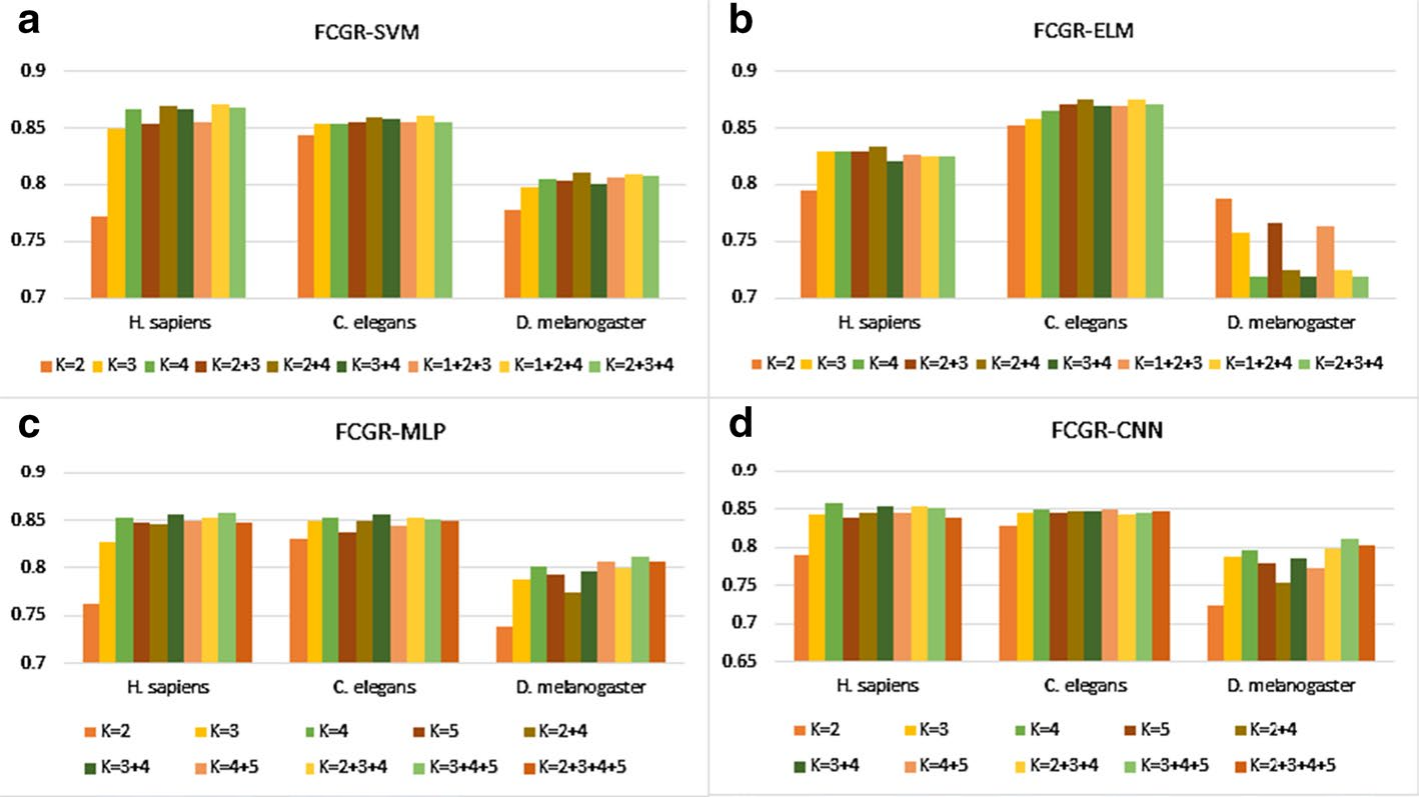
The histogram (**a**–**d**) shows the accuracy of using SVM, ELM, MLP and CNN with K = 1, 2, 3, 4, 5 or combinations

In this work, we flatten the FCGR matrix into a normalized vector (1-D) corresponding to the frequency of K nucleotides as the input of SVM and ELM [27]. The input of MLP and CNN models are not only single-channel FCGR images (2D) [26, 27], but also multiple K-value images, the image size is 64 × 64. For the input of multi-K-value images, we leveraged multiple channels to feed in the combination of K values when training the model, and used simple averaging to calculate the final prediction result. To find the appropriate value of K or combination, we use 10-fold cross-validation. Figure 1 shows the classification accuracy of each classifier with different K values and combinations.

For SVM, the accuracy of H. sapiens, C. elegans reaches its peak with K = 1, 2 and 4; the accuracy of D. melanogaster was the highest with K = 2 and 4. For ELM, the accuracy of D. melanogaster reaches an peak when K = 2; the accuracy of H. sapiens reaches its peak when K = 2 and 4; the classification accuracy of C. elegans is best with K = 1, 2 and 4 like using SVM.

For MLP, the accuracy of H. sapiens and D. melanogaster reaches its peak with K = 3, 4 and 5; the classification accuracy of C. elegans is best with K = 3 and 4. For CNN, H. sapiens have the best classification quality when using the FCGR image with K = 4; the accuracy of C. elegans reaches its peak with K = 4 and 5; the accuracy of D. melanogaster reaches its peak with K = 3, 4 and 5. Table 1 clearly shows the best prediction results for four species via 10-fold cross-validation.

**Table 1:** The prediction results for four species via 10-fold cross-validation by SVM, ELM, MLP, CNN

Species	Method	K	ACC	Sn	Sp	MCC	AUC
H. sapiens	FCGR-SVM	1 + 2 + 4	**0.8708**	**0.8980**	**0.8439**	**0.7432**	**0.9300**
	FCGR-ELM	2 + 4	0.8332	0.8773	0.7896	0.6695	0.8969
	FCGR-MLP	3 + 4 + 5	0.8565	0.8768	0.8365	0.7144	0.9186
	FCGR-CNN	4	0.8585	0.8746	0.8426	0.7185	0.9214
C. elegans	FCGR-SVM	1 + 2 + 4	0.8603	0.8948	0.8263	0.7229	0.9295
	FCGR-ELM	1 + 2 + 4	**0.8754**	**0.8944**	**0.8566**	**0.7515**	**0.9421**
	FCGR-MLP	3 + 4	0.8537	0.8613	0.8462	0.7092	0.9225
	FCGR-CNN	4 + 5	0.8495	0.8839	0.8156	0.702	0.9181
D. melanogaster	FCGR-SVM	2 + 4	**0.8113**	**0.7831**	**0.8400**	**0.6241**	**0.8791**
	FCGR-ELM	2	0.7910	0.7648	0.8175	0.5833	0.8595
	FCGR-MLP	3 + 4 + 5	0.8117	0.8000	0.8235	0.6238	0.8848
	FCGR-CNN	3 + 4 + 5	0.8108	0.8014	0.8204	0.6228	0.8854
S. cerevisiae	FCGR-SVM	4	**1**	**1**	**1**	**1**	**1**
	FCGR-ELM	3 or 4	**1**	**1**	**1**	**1**	**1**
	FCGR-MLP	4	**1**	**1**	**1**	**1**	**1**
	FCGR-CNN	4	0.9997	1	0.9994	0.9995	1

Best values are in bold

For S. cerevisiae dataset, we used SVM, ELM and MLP to achieve $S_{n}$ = $S_{p}$ = ACC = MCC = AUC = 1 via 10-fold cross-validation when K = 3 or 4. There may be room for improvement in the predicted quality of the other three datasets.

### Comparison of the results with integrative features

In addition, we also integrated FCGR with other feature representations [29–32], such as DAC, TAC, DACC, TACC, PC-PseDNC, PC-PseTNC, and input them into SVM and ELM. Besides, we added the extreme gradient boosting (XGBoost) algorithm. The comparative analysis results are shown in Tables 2, 3 and 4 respectively.

From the results in Tables 2, 3 and 4, the combination of FCGR and DAC as feature vectors have a greater prediction quality. XGBoost performance is relatively stable, and each prediction results have little difference, especially for inputting high-dimensional features. However, after some high-dimensional feature vectors are input into SVM and ELM, the prediction results are relatively poor. It shows that XGBoost is more suitable for processing high-dimensional features.

**Table 2:** The prediction results of integrative feature representation for H. sapiens via 10-fold cross-validation by SVM, ELM and XGBoost

Method	Feature	parameter	ACC	Sn	Sp	MCC	AUC
SVM	FCGR + DAC	K = 4, lag = 2	**0.8708**	0.8896	0.8522	0.7425	**0.9315**
	FCGR + TAC	K = 4, lag = 2	0.8679	0.8878	0.8483	0.7369	0.9288
	FCGR + DACC	K = 4, lag = 2	0.8537	0.8531	0.8544	0.7079	0.9208
	FCGR + TACC	K = 4, lag = 2	0.8415	0.8319	0.8509	0.6837	0.9113
	FCGR + PCPseDNC	K = 4, λ = 8, w = 0.5	0.8673	0.8936	0.8413	0.7359	0.9286
	FCGR + PCPseTNC	K = 4, λ = 8, w = 0.5	0.8708	**0.8966**	0.8452	**0.7429**	0.9273
	All features		0.8137	0.7518	**0.8748**	0.6322	0.8996
ELM	FCGR + DAC	K = 4, lag = 2	0.8292	0.8539	0.8048	0.6598	0.9007
	FCGR + TAC	K = 4, lag = 2	0.8297	0.8531	0.8065	0.6604	0.8977
	FCGR + DACC	K = 4, lag = 2	0.8336	0.8627	0.8048	0.6689	0.9009
	FCGR + TACC	K = 4, lag = 2	0.8325	0.8632	0.8022	0.6668	0.8983
	FCGR + PCPseDNC	K = 4, λ = 8, w = 0.5	0.8314	0.8658	0.7974	0.6648	0.8985
	FCGR + PCPseTNC	K = 4, λ = 8, w = 0.5	0.8248	0.8544	0.7957	0.6516	0.8947
	All features		0.8356	0.8632	0.8083	0.6735	0.9013
XGBoost	FCGR	K = 1 + 2 + 4	0.8585	0.89309	0.8244	0.71934	0.9197
	FCGR + DAC	K = 4, lag = 2	0.8450	0.87503	0.8152	0.69182	0.9160
	FCGR + TAC	K = 4, lag = 2	0.8402	0.8733	0.8074	0.68221	0.9136
	FCGR + DACC	K = 4, lag = 2	0.8423	0.86583	0.8191	0.68588	0.9127
	FCGR + TACC	K = 4, lag = 2	0.8391	0.87287	0.8057	0.68059	0.9115
	FCGR + PCPseDNC	K = 4, λ = 8, w = 0.5	0.8559	0.88913	0.8230	0.71396	0.9207
	FCGR + PCPseTNC	K = 4, λ = 8, w = 0.5	0.8498	0.88254	0.8174	0.70168	0.9183
	All features		0.8472	0.87374	0.8209	0.69581	0.9170

All features means the feature vector = FCGR + DACC + TACC + PC-PseDNC + PC-pseTAC, and the parameters are consistent with the parameters of the corresponding feature. Parameter K indicates the values of K nucleotide in FCGR; lag indicates the distance of lag along the sequence; λ represents the highest counted rank (or tier) of the correlation along a DNA sequence; w is the weight factor ranged from 0 to 1

Best values are in bold

**Table 3:** The prediction results of integrative feature representation for C. elegans via 10-fold cross-validation by SVM, ELM and XGBoost

Method	Feature	parameter	ACC	Sn	Sp	MCC	AUC
SVM	FCGR + DAC	K = 4, lag = 2	0.8574	0.8863	0.8290	0.7164	0.9283
	FCGR + TAC	K = 4, lag = 2	0.8561	0.8824	0.8302	0.7137	0.9272
	FCGR + DACC	K = 4, lag = 2	0.8471	0.8777	0.8171	0.6961	0.9122
	FCGR + TACC	K = 4, lag = 2	0.8470	0.8641	0.8301	0.6949	0.9179
	FCGR + PCPseDNC	K = 4, λ = 8, w = 0.5	0.8576	**0.8921**	0.8236	0.7176	0.9275
	FCGR + PCPseTNC	K = 4, λ = 8, w = 0.5	0.8539	0.8839	0.8244	0.7096	0.9275
	All features		0.8431	0.8461	0.8401	0.6867	0.9139
ELM	FCGR + DAC	K = 4, lag = 2	**0.8707**	0.8863	**0.8555**	**0.7421**	0.9355
	FCGR + TAC	K = 4, lag = 2	0.8696	0.8890	0.8505	0.7400	**0.9359**
	FCGR + DACC	K = 4, lag = 2	0.8684	0.8831	0.8539	0.7376	0.9358
	FCGR + TACC	K = 4, lag = 2	0.8680	0.8917	0.8447	0.7371	0.9329
	FCGR + PCPseDNC	K = 4, λ = 8, w = 0.5	0.8624	0.8847	0.8405	0.7258	0.9318
	FCGR + PCPseTNC	K = 4, λ = 8, w = 0.5	0.8557	0.8847	0.8271	0.7132	0.9262
	All features		0.8597	0.8863	0.8336	0.7210	0.9271
XGBoost	FCGR	K = 1 + 2 + 4	0.8487	0.8797	0.8182	0.6995	0.9202
	FCGR + DAC	K = 4, lag = 2	0.8416	0.8652	0.8182	0.6842	0.9165
	FCGR + TAC	K = 4, lag = 2	0.8433	0.8707	0.8163	0.6882	0.9169
	FCGR + DACC	K = 4, lag = 2	0.8462	0.8703	0.8225	0.6938	0.9170
	FCGR + TACC	K = 4, lag = 2	0.8417	0.8676	0.8163	0.6848	0.9162
	FCGR + PCPseDNC	K = 4, λ = 8, w = 0.5	0.8450	0.8749	0.8156	0.6917	0.9199
	FCGR + PCPseTNC	K = 4, λ = 8, w = 0.5	0.8493	0.8789	0.8202	0.7004	0.9178
	All features		0.8481	0.8695	0.8271	0.6973	0.9195

All features means the feature vector = FCGR + DACC + TACC + PC-PseDNC + PC-pseTAC, and the parameters are consistent with the parameters of the corresponding feature. Parameter K indicates the values of K nucleotide in FCGR; lag indicates the distance of lag along the sequence; λ represents the highest counted rank (or tier) of the correlation along a DNA sequence; w is the weight factor ranged from 0 to 1

Best values are in bold

**Table 4:** The prediction results of integrative feature representation for D. melanogaster via 10-fold cross-validation by SVM, ELM and XGBoost

Method	Feature	parameter	ACC	Sn	Sp	MCC	AUC
SVM	FCGR + DAC	K = 4, lag = 2	0.8047	**0.7862**	0.8235	0.6103	0.8762
	FCGR + TAC	K = 4, lag = 2	**0.8089**	0.7835	0.8347	**0.6190**	0.8747
	FCGR + DACC	K = 4, lag = 2	0.7753	0.7772	0.7733	0.5509	0.8295
	FCGR + TACC	K = 4, lag = 2	0.7560	0.6772	**0.8361**	0.5199	0.8247
	FCGR + PCPseDNC	K = 4, λ = 8, w = 0.5	0.8073	0.7797	0.8354	0.6162	**0.8803**
	FCGR + PCPseTNC	K = 4, λ = 8, w = 0.5	0.8057	0.7835	0.8284	0.6129	0.8769
	All features		0.7510	0.6828	0.8204	0.5078	0.7987
ELM	FCGR + DAC	K = 2, lag = 2	0.7920	0.7779	0.8063	0.5847	0.8644
	FCGR + TAC	K = 2, lag = 2	0.7917	0.7807	0.8028	0.5839	0.8651
	FCGR + DACC	K = 2, lag = 2	0.7769	0.7617	0.7923	0.5544	0.8503
	FCGR + TACC	K = 2, lag = 2	0.7694	0.7735	0.7653	0.5391	0.8460
	FCGR + PCPseDNC	K = 2, λ = 8, w = 0.5	0.7896	0.7631	0.8165	0.5806	0.8651
	FCGR + PCPseTNC	K = 2, λ = 8, w = 0.5	0.7595	0.7341	0.7853	0.5206	0.8400
	All features		0.7847	0.7810	0.7884	0.5700	0.8576
XGBoost	FCGR	K = 1 + 2 + 4	0.7976	0.7797	0.8158	0.5959	0.8725
	FCGR + DAC	K = 4, lag = 2	0.7873	0.7717	0.8032	0.5751	0.8613
	FCGR + TAC	K = 4, lag = 2	0.7877	0.7624	0.8133	0.5768	0.8647
	FCGR + DACC	K = 4, lag = 2	0.7724	0.7814	0.7632	0.5450	0.8532
	FCGR + TACC	K = 4, lag = 2	0.7824	0.7693	0.7958	0.5658	0.8542
	FCGR + PCPseDNC	K = 4, λ = 8, w = 0.5	0.7997	0.7824	0.8172	0.6001	0.8725
	FCGR + PCPseTNC	K = 4, λ = 8, w = 0.5	0.7988	0.7790	0.8190	0.5989	0.8775
	All features		0.7951	0.7793	0.8112	0.5909	0.8718

All features means the feature vector = FCGR + DACC + TACC + PC-PseDNC + PC-pseTAC, and the parameters are consistent with the parameters of the corresponding feature. Parameter K indicates the values of K nucleotide in FCGR; lag indicates the distance of lag along the sequence; λ represents the highest counted rank (or tier) of the correlation along a DNA sequence; w is the weight factor ranged from 0 to 1

Best values are in bold

### Comparison of the results with dimensionality reduction

**Fig. 2:**
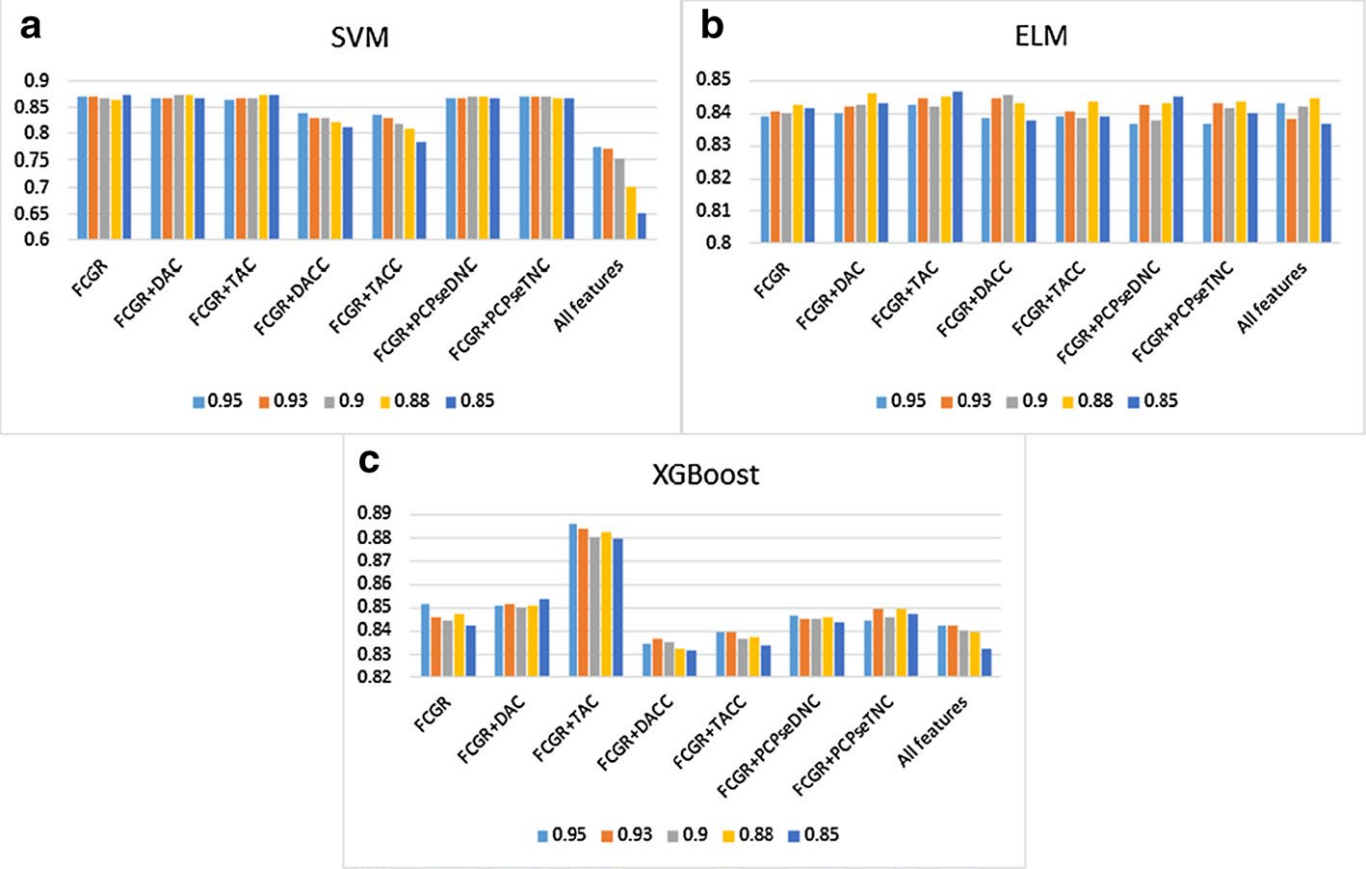
The histogram (**a**–**c**) shows the accuracy of using SVM, ELM and XGBoost with contributing rate of principal component = 0.95, 0.93, 0.9, 0.88, 0.85 for H. sapiens

**Fig. 3:**
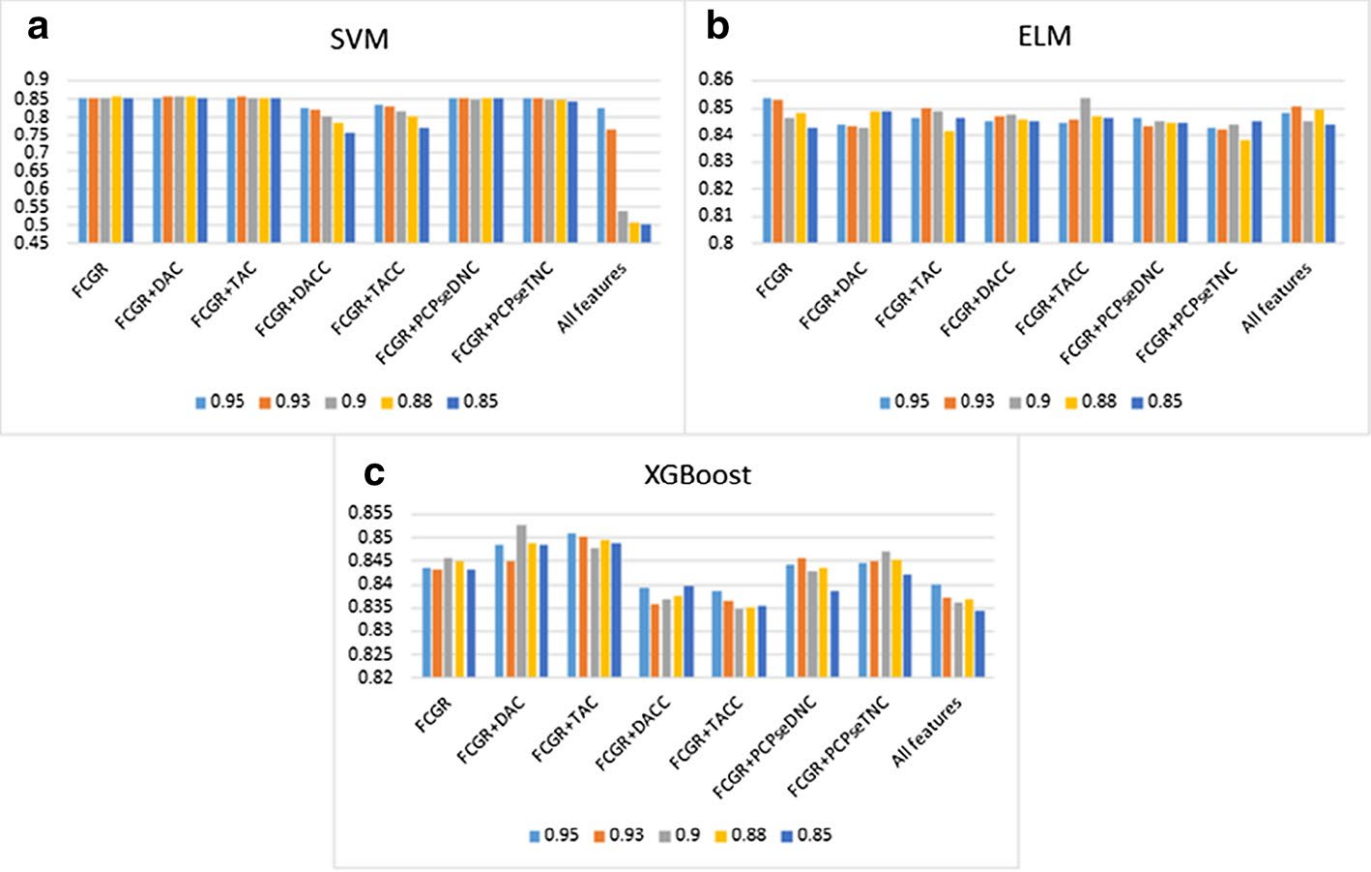
The histogram (**a**–**c**) shows the accuracy of using SVM, ELM and XGBoost with contributing rate of principal component = 0.95, 0.93, 0.9, 0.88, 0.85 for C. elegans

**Fig. 4:**
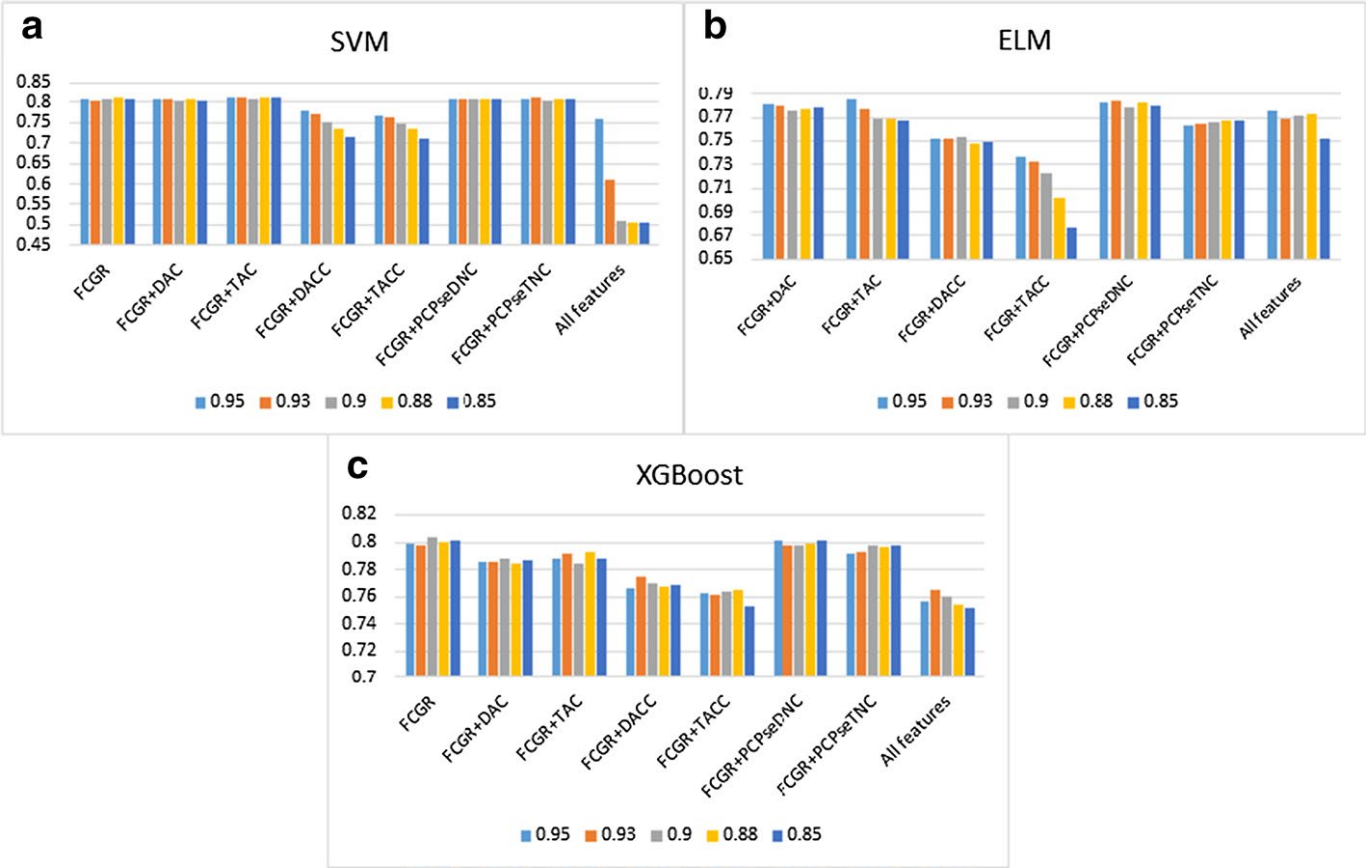
The histogram (**a**–**c**) shows the accuracy of using SVM, ELM and XGBoost with contributing rate of principal component = 0.95, 0.93, 0.9, 0.88, 0.85 for D. melanogaster

Considering the high dimensionality of the integrative feature vector, it is possible that high-dimensional feature vectors would bring the curse of dimensionality, which leads to overfitting of the prediction result. Therefore, we also adopted the principal component analysis (PCA) algorithm [33] to achieve feature dimensionality reduction. Then, the feature vector after dimensionality reduction is input into SVM, ELM and XGBoost respectively. In the process of using PCA to dimensionality reduction, the cumulative contribution rate of the retained principal components will directly affect the dimensionality reduction effect. Therefore, we calculated the accuracy of 95%, 93%, 90%, 88% and 85% of the contribution rate of the retained principal components respectively. Figures 2, 3 and 4 shows the classification accuracy of each classifier with different contributing rate of principal component. And the results of the optimal contribution rate of the principal components corresponding to each predictor are shown in Tables 5, 6 and 7 respectively.

From Tables 5, 6 and 7, We have noticed that the prediction quality has been improved after dimensionality reduction through PCA for H. sapiens. It is increased by 4.57%, 3.12%, 6.00%, 9.03%, 3.56% in ACC, $S_{n}$, $S_{p}$, MCC and AUC when we combined FCGR vectors and TAC for using with XGBoost. However, the prediction quality has been not improved significantly for C. elegans. Especially when ELM was used, its prediction quality decreased slightly. For D. melanogaster, similarly, there is no significant improvement.

**Table 5:** PCA dimensionality reduction results via 10-fold cross-validation for H. sapiens

Method	Feature	Parameters	PCA%	ACC	Sn	Sp	MCC	AUC
SVM	FCGR	K = 1 + 2 + 4	0.85	**0.8758**	**0.8966**	0.8552	**0.7528**	0.9288
	FCGR + DAC	K = 4, lag = 2	0.9	0.8749	0.8856	**0.8644**	0.7507	**0.9314**
	FCGR + TAC	K = 4, lag = 2	0.85	0.8752	0.8878	0.8626	0.7513	0.9306
	FCGR + DACC	K = 4, lag = 2	0.95	0.8410	0.8236	0.8583	0.6825	0.9138
	FCGR + TACC	K = 4, lag = 2	0.95	0.8369	0.8170	0.8565	0.6749	0.9099
	FCGR + PCPseDNC	K = 4, λ = 8, w = 0.5	0.88	0.8727	0.8896	0.8561	0.7463	0.9284
	FCGR + PCPseTNC	K = 4, λ = 8, w = 0.5	0.9	0.8719	0.8878	0.8561	0.7444	0.9281
	All features		0.95	0.7746	0.6388	0.9087	0.5698	0.8906
ELM	FCGR	K = 1 + 2 + 4	0.88	0.8428	0.8636	0.8222	0.6866	0.9075
	FCGR + DAC	K = 4, lag = 2	0.88	0.8461	0.8724	0.8200	**0.6936**	0.9128
	FCGR + TAC	K = 4, lag = 2	0.85	**0.8469**	0.8698	0.8244	0.6952	**0.9129**
	FCGR + DACC	K = 4, lag = 2	0.9	0.8458	0.8763	0.8157	0.6936	0.9095
	FCGR + TACC	K = 4, lag = 2	0.88	0.8439	0.8808	0.8074	0.6902	0.9072
	FCGR + PCPseDNC	K = 4, λ = 8, w = 0.5	0.85	0.8454	0.8645	**0.8265**	0.6918	0.9107
	FCGR + PCPseTNC	K = 4, λ = 8, w = 0.5	0.88	0.8437	0.8627	0.8248	0.6882	0.9069
	All features		0.88	0.8447	**0.8843**	0.8057	0.6923	0.9118
XGBoost	FCGR	K = 1 + 2 + 4	0.95	0.8513	0.8733	0.8296	0.7037	0.9175
	FCGR + DAC	K = 4, lag = 2	0.85	0.8537	0.8667	0.8409	0.7080	0.9172
	FCGR + TAC	K = 4, lag = 2	0.95	**0.8859**	**0.9045**	**0.8674**	**0.7725**	**0.9491**
	FCGR + DACC	K = 4, lag = 2	0.93	0.8364	0.8601	0.8130	0.6741	0.9014
	FCGR + TACC	K = 4, lag = 2	0.95	0.8395	0.8667	0.8126	0.6805	0.9050
	FCGR + PCPseDNC	K = 4, λ = 8, w = 0.5	0.95	0.8463	0.8711	0.8217	0.6937	0.9147
	FCGR + PCPseTNC	K = 4, λ = 8, w = 0.5	0.93	0.8498	0.8645	0.8352	0.7003	0.9155
	All features		0.95	0.8423	0.8729	0.8122	0.6864	0.9051

“PCA%” means contributing rate of principal component

Best values are in bold

**Table 6:** PCA dimensionality reduction results via 10-fold cross-validation for C. elegans

Method	Feature	Parameters	PCA%	ACC	Sn	Sp	MCC	AUC
SVM	FCGR	K = 1 + 2 + 4	0.88	0.8551	0.8960	0.8148	0.7130	0.9242
	FCGR + DAC	K = 4, lag = 2	0.9	**0.8562**	0.8870	0.8259	**0.7142**	**0.9245**
	FCGR + TAC	K = 4, lag = 2	0.93	0.8558	0.8824	0.8297	0.7132	**0.9245**
	FCGR + DACC	K = 4, lag = 2	0.95	0.8265	**0.9147**	0.7397	0.6642	0.9057
	FCGR + TACC	K = 4, lag = 2	0.95	0.8336	0.8079	0.8589	0.6682	0.9052
	FCGR + PCPseDNC	K = 4, λ = 8, w = 0.5	0.85	0.8543	0.8913	0.8179	0.7112	0.9236
	FCGR + PCPseTNC	K = 4, λ = 8, w = 0.5	0.95	0.8516	0.8929	0.8110	0.7064	0.9243
	All features		0.95	0.8249	0.8029	**0.8466**	0.6513	0.8823
ELM	FCGR	K = 1 + 2 + 4	0.95	0.8535	**0.8882**	0.8194	**0.7093**	**0.9193**
	FCGR + DAC	K = 4, lag = 2	0.88	0.8489	0.8742	0.8240	0.6990	0.9124
	FCGR + TAC	K = 4, lag = 2	0.93	0.8500	0.8742	**0.8263**	0.7012	0.9157
	FCGR + DACC	K = 4, lag = 2	0.9	0.8476	0.8703	0.8252	0.6962	0.9159
	FCGR + TACC	K = 4, lag = 2	0.9	**0.8537**	0.8808	0.8271	0.7090	0.9183
	FCGR + PCPseDNC	K = 4, λ = 8, w = 0.5	0.95	0.8466	0.8777	0.8160	0.6951	0.9158
	FCGR + PCPseTNC	K = 4, λ = 8, w = 0.5	0.85	0.8452	0.8679	0.8229	0.6915	0.9160
	All features		0.93	0.8505	0.8816	0.8198	0.7030	0.9183
XGBoost	FCGR	K = 1 + 2 + 4	0.90	0.8458	**0.8870**	0.8052	0.6946	0.9175
	FCGR + DAC	K = 4, lag = 2	0.90	**0.8526**	0.8831	0.8225	**0.7068**	**0.9234**
	FCGR + TAC	K = 4, lag = 2	0.95	0.8508	0.8738	**0.8282**	0.7028	0.9195
	FCGR + DACC	K = 4, lag = 2	0.85	0.8396	0.8570	0.8225	0.6800	0.9147
	FCGR + TACC	K = 4, lag = 2	0.95	0.8385	0.8621	0.8152	0.6782	0.9110
	FCGR + PCPseDNC	K = 4, λ = 8, w = 0.5	0.93	0.8456	0.8808	0.8110	0.6934	0.9200
	FCGR + PCPseTNC	K = 4, λ = 8, w = 0.5	0.90	0.8472	0.8835	0.8114	0.6967	0.9191
	All features		0.95	0.8400	0.8613	0.8190	0.6812	0.9143

“PCA%” means contributing rate of principal component

Best values are in bold

**Table 7:** PCA dimensionality reduction results via 10-fold cross-validation for D. melanogaster

Method	Feature	Parameters	PCA%	ACC	Sn	Sp	MCC	AUC
SVM	FCGR	K = 1 + 2 + 4	0.88	**0.8108**	0.7786	0.8435	**0.6235**	**0.8785**
	FCGR + DAC	K = 4, lag = 2	0.95	0.8070	0.7855	0.8288	0.6152	0.8768
	FCGR + TAC	K = 4, lag = 2	0.93	0.8115	0.7831	0.8404	0.6245	0.8766
	FCGR + DACC	K = 4, lag = 2	0.95	0.7809	**0.7931**	0.7684	0.5621	0.8343
	FCGR + TACC	K = 4, lag = 2	0.95	0.7678	0.6879	**0.8491**	0.5440	0.8363
	FCGR + PCPseDNC	K = 4, λ = 8, w = 0.5	0.9	0.8085	0.7752	0.8425	0.6190	0.8773
	FCGR + PCPseTNC	K = 4, λ = 8, w = 0.5	0.93	0.8097	0.7772	0.8428	0.6215	0.8761
	All features		0.95	0.7593	0.70414	0.81544	0.52275	0.80283
ELM	FCGR + DAC	K = 2, lag = 2	0.95	0.7817	0.7690	0.7947	0.5642	0.8544
	FCGR + TAC	K = 2, lag = 2	0.95	**0.7859**	**0.7735**	0.7986	0.5723	0.8552
	FCGR + DACC	K = 2, lag = 2	0.9	0.7530	0.7524	0.7537	0.5064	0.8262
	FCGR + TACC	K = 2, lag = 2	0.95	0.7365	0.7472	0.7256	0.4733	0.8018
	FCGR + PCPseDNC	K = 2, λ = 8, w = 0.5	0.93	0.7837	0.7597	**0.8081**	**0.5685**	**0.8587**
	FCGR + PCPseTNC	K = 2, λ = 8, w = 0.5	0.88	0.7678	0.7283	**0.8081**	0.5379	0.8448
	All features	K = 2	0.95	0.7727	0.7714	0.7740	0.5455	0.8437
XGBoost	FCGR	K = 1 + 2 + 4	0.9	**0.8037**	**0.7824**	**0.8253**	**0.6085**	**0.8772**
	FCGR + DAC	K = 4, lag = 2	0.9	0.7877	0.7683	0.8074	0.5763	0.8630
	FCGR + TAC	K = 4, lag = 2	0.88	0.7930	0.7635	0.8232	0.5879	0.8671
	FCGR + DACC	K = 4, lag = 2	0.93	0.7741	0.7690	0.7793	0.5486	0.8506
	FCGR + TACC	K = 4, lag = 2	0.88	0.7654	0.7576	0.7733	0.5313	0.8461
	FCGR + PCPseDNC	K = 4, λ = 8, w = 0.5	0.88	0.7988	0.7769	0.8211	0.5987	0.8753
	FCGR + PCPseTNC	K = 4, λ = 8, w = 0.5	0.85	0.7974	0.7772	0.8179	0.5960	0.8727
	All features		0.93	0.7647	0.7590	0.7705	0.5295	0.8406

“PCA%” means contributing rate of principal component

Best values are in bold

### Comparison with other algorithms

To verify the effectiveness of our method, we compared the prediction results of the optimal performing predictors in Tables 1, 2, 3 and 4 with other models using the same datasets. DLNN-5 [24] is a deep learning model with a convolution kernel size of 5, and ZCMM [23] is based on SVM. Tables 8, 9, 10 and 11 shows that our methods perform prominently on H. sapiens and S. cerevisiae datasets. For S. cerevisiae dataset, we used SVM, ELM and MLP to achieve $S_{n} = S_{p}$ = ACC = MCC = AUC = 1 via 10-fold cross-validation when K = 3 or 4. Compared with the model that based on DNA deformation energy in the original paper [20], the prediction performance has been obviously lifted. For H. sapiens, combined FCGR vectors and TAC for using with XGBoost is higher than ZCMM in ACC, $S_{n}$, $S_{p}$, MCC, AUC by 10.87%, 15.58%, 5.23%, 21.25%, 8.81%, respectively; likewise, it is higher than DLNN-5 in ACC, $S_{n}$, $S_{p}$ by 3.22%, 2.11%, 4.45%, respectively. The performance of CNN is slightly better than ZCMM and DLNN-5. For C. elegans, compared with ZCMM, we use ELM to increase the evaluation indicators by 2.20%, 10.64%, 1.56%, 13.15%, 3.01% when combined FCGR vectors with K = 1, 2 and 4. For D. melanogaster, our prediction accuracy is lower, and ZCMM's prediction accuracy (ACC) is the highest at 93.62%. Results imply that our final prediction is positive, it only performed unfavorably on the D. melanogaster dataset.

**Table 8:** Comparison of our predictors with other models via 10-fold cross-validation for S. cerevisiae

Method	ACC	Sn	Sp	MCC	AUC
Deformation energy [20]	0.981	0.982	0.980	0.963	~
FCGR-SVM	1	1	1	1	1
FCGR-ELM	1	1	1	1	1
FCGR-MLP	1	1	1	1	1
FCGR-CNN	0.9997	1	0.9994	0.9995	1

**Table 9:** Comparison of our predictors with other models via 10-fold cross-validation for H. sapiens

Method	Feature	ACC	Sn	Sp	MCC	AUC
DLNN-5 [24]		0.8537	0.8834	0.8229	~	~
ZCMM [23]		0.7772	0.7487	0.8151	0.5600	0.8610
SVM	FCGR	0.8758	0.8966	0.8552	0.7528	0.9288
	FCGR + DAC	0.8749	0.8856	0.8644	0.7507	0.9314
	FCGR + TAC	0.8752	0.8878	0.8626	0.7513	0.9306
	FCGR + DACC	0.8537	0.8531	0.8544	0.7079	0.9208
	FCGR + TACC	0.8415	0.8319	0.8509	0.6837	0.9113
	FCGR + PCPseDNC	0.8727	0.8896	0.8561	0.7463	0.9284
	FCGR + PCPseTNC	0.8719	0.8878	0.8561	0.7444	0.9281
	All features	0.8137	0.7518	0.8748	0.6322	0.8996
ELM	FCGR	0.8428	0.8636	0.8222	0.6866	0.9075
	FCGR + DAC	0.8461	0.8724	0.8200	0.6936	0.9128
	FCGR + TAC	0.8469	0.8698	0.8244	0.6952	0.9129
	FCGR + DACC	0.8458	0.8763	0.8157	0.6936	0.9095
	FCGR + TACC	0.8439	0.8808	0.8074	0.6902	0.9072
	FCGR + PCPseDNC	0.8454	0.8645	0.8265	0.6918	0.9107
	FCGR + PCPseTNC	0.8437	0.8627	0.8248	0.6882	0.9069
	All features	0.8447	0.8843	0.8057	0.6923	0.9118
XGBoost	FCGR	0.8585	0.89309	0.8244	0.71934	0.9197
	FCGR + DAC	0.8537	0.8667	0.8409	0.708	0.9172
	FCGR + TAC	**0.8859**	**0.9045**	**0.8674**	**0.7725**	**0.9491**
	FCGR + DACC	0.8423	0.86583	0.8191	0.68588	0.9127
	FCGR + TACC	0.8395	0.8667	0.8126	0.6805	0.905
	FCGR + PCPseDNC	0.8559	0.88913	0.823	0.71396	0.9207
	FCGR + PCPseTNC	0.8498	0.8645	0.8352	0.7003	0.9155
	All features	0.8472	0.87374	0.8209	0.69581	0.917
MLP	FCGR	0.8565	0.8768	0.8365	0.7144	0.9186
CNN	FCGR	0.8585	0.8746	0.8426	0.7185	0.9214

The table shows the optimal results of each classifier, and the specific parameters are shown in the previous tables

Best values are in bold

**Table 10:** Comparison of our predictors with other models via 10-fold cross-validation for C. elegans

Method	Feature	ACC	Sn	Sp	MCC	AUC
DLNN-5 [24]		**0.8962**	**0.9304**	**0.8634**	** ~ **	** ~ **
ZCMM [23]		0.8534	0.7880	0.8410	0.6200	0.9120
SVM	FCGR	0.8603	0.8948	0.8263	0.7229	0.9295
	FCGR + DAC	0.8574	0.8863	0.8290	0.7164	0.9283
	FCGR + TAC	0.8561	0.8824	0.8302	0.7137	0.9272
	FCGR + DACC	0.8471	0.8777	0.8171	0.6961	0.9122
	FCGR + TACC	0.8470	0.8641	0.8301	0.6949	0.9179
	FCGR + PCPseDNC	0.8576	0.8921	0.8236	0.7176	0.9275
	FCGRPCPseTNC	0.8539	0.8839	0.8244	0.7096	0.9275
	All features	0.8431	0.8461	0.8401	0.6867	0.9139
ELM	FCGR	0.8754	0.8944	0.8566	0.7515	0.9421
	FCGR + DAC	0.8707	0.8863	0.8555	0.7421	0.9355
	FCGR + TAC	0.8696	0.8890	0.8505	0.7400	0.9359
	FCGR + DACC	0.8684	0.8831	0.8539	0.7376	0.9358
	FCGR + TACC	0.8680	0.8917	0.8447	0.7371	0.9329
	FCGR + PCPseDNC	0.8624	0.8847	0.8405	0.7258	0.9318
	FCGR + PCPseTNC	0.8557	0.8847	0.8271	0.7132	0.9262
	All features	0.8597	0.8863	0.8336	0.7210	0.9271
XGBoost	FCGR	0.8487	0.8797	0.8182	0.6995	0.9202
	FCGR + DAC	0.8526	0.8831	0.8225	0.7068	0.9234
	FCGR + TAC	0.8508	0.8738	0.8282	0.7028	0.9195
	FCGR + DACC	0.8462	0.8703	0.8225	0.6938	0.917
	FCGR + TACC	0.8417	0.8676	0.8163	0.6848	0.9162
	FCGR + PCPseDNC	0.8456	0.8808	0.811	0.6934	0.92
	FCGR + PCPseTNC	0.8493	0.8789	0.8202	0.7004	0.9178
	All features	0.8481	0.8695	0.8271	0.6973	0.9195
MLP	FCGR	0.8537	0.8613	0.8462	0.7092	0.9225
CNN	FCGR	0.8495	0.8839	0.8156	0.702	0.9181

The table shows the optimal results of each classifier, and the specific parameters are shown in the previous tables

Best values are in bold

**Table 11:** Comparison of our predictors with other models via 10-fold cross-validation for D. melanogaster

Method	Feature	ACC	Sn	Sp	MCC	AUC
DLNN-5 [24]		0.8560	0.8781	0.8333	~	~
ZCMM [23]		**0.9362**	**0.9226**	**0.7964**	**0.7000**	**0.9110**
SVM	FCGR	0.8113	0.7831	0.84	0.6241	0.8791
	FCGR + DAC	0.8089	0.7835	0.8347	0.619	0.8747
	FCGR + TAC	0.8115	0.7831	0.8404	0.6245	0.8766
	FCGR + DACC	0.7809	0.7931	0.7684	0.5621	0.8343
	FCGR + TACC	0.7678	0.6879	0.8491	0.544	0.8363
	FCGR + PCPseDNC	0.8085	0.7752	0.8425	0.619	0.8773
	FCGR + PCPseTNC	0.8097	0.7772	0.8428	0.6215	0.8761
	All features	0.7593	0.70414	0.81544	0.52275	0.80283
ELM	FCGR	0.791	0.7648	0.8175	0.5833	0.8595
	FCGR + DAC	0.792	0.7779	0.8063	0.5847	0.8644
	FCGR + TAC	0.7917	0.7807	0.8028	0.5839	0.8651
	FCGR + DACC	0.7769	0.7617	0.7923	0.5544	0.8503
	FCGR + TACC	0.7694	0.7735	0.7653	0.5391	0.846
	FCGR + PCPseDNC	0.7896	0.7631	0.8165	0.5806	0.8651
	FCGR + PseTNC	0.7678	0.7283	0.8081	0.5379	0.8448
	All features	0.7847	0.781	0.7884	0.57	0.8576
XGBoost	FCGR	0.8037	0.7824	0.8253	0.6085	0.8772
	FCGR + DAC	0.7877	0.7683	0.8074	0.5763	0.863
	FCGR + TAC	0.793	0.7635	0.8232	0.5879	0.8671
	FCGR + DACC	0.7741	0.769	0.7793	0.5486	0.8506
	FCGR + TACC	0.7824	0.7693	0.7958	0.5658	0.8542
	FCGR + PCPseDNC	0.7997	0.7824	0.8172	0.6001	0.8725
	FCGR + PseTNC	0.7988	0.779	0.819	0.5989	0.8775
	All features	0.7951	0.7793	0.8112	0.5909	0.8718
MLP	FCGR	0.8117	0.8000	0.8235	0.6238	0.8848
CNN	FCGR	0.8108	0.8014	0.8204	0.6228	0.8854

The table shows the optimal results of each classifier, and the specific parameters are shown in the previous tables

Best values are in bold

### Comparison with other advanced methods

In addition to DLNN-5 and ZCMM models, there are some other advanced methods for nucleosome prediction in the same dataset. LeNup model utilizes improved convolutional neural networks, which adds inception modules and gated convolutional networks [25]. 3LS is based on the linear regression model [22]. LeNup used the 20-fold cross-validation and provided comparison data with 3LS for H. sapiens, C. elegans and D. melanogaster. Therefore, we utilized the results provided by LeNup for comparative analysis in Tables 12, 13 and 14.

**Table 12:** Comparison of our predictors with other advanced models via 20-fold cross-validation for H. sapiens

Method	Feature	ACC	Sn	Sp	MCC	AUC
LeNup [25]		0.8889	0.9212	0.8562	0.7906	0.9412
3LS [22]		**0.9001**	**0.9169**	**0.8835**	**0.8006**	**0.9588**
SVM	FCGR	0.8760	0.8940	0.8583	0.7535	0.9288
	FCGR + DAC	0.8751	0.8874	0.8630	0.7513	0.9310
	FCGR + TAC	0.8754	0.8869	0.8639	0.7519	0.9318
	FCGR + DACC	0.8563	0.8544	0.8583	0.7138	0.9217
	FCGR + TACC	0.8423	0.8337	0.8509	0.6858	0.9114
	FCGR + PseDNC	0.8736	0.8883	0.8591	0.7481	0.9294
	FCGR + PseTNC	0.8740	0.8905	0.8578	0.7491	0.9280
	All features	0.8154	0.7545	0.8757	0.6355	0.8998
ELM	FCGR	0.8456	0.8702	0.8213	0.6931	0.9092
	FCGR + DAC	0.8469	0.8707	0.8235	0.6952	0.9087
	FCGR + TAC	0.8478	0.8750	0.8209	0.6974	0.9142
	FCGR + DACC	0.8500	0.8772	0.8230	0.7017	0.9054
	FCGR + TACC	0.8454	0.8865	0.8048	0.6941	0.9104
	FCGR + PseDNC	0.8476	0.8640	0.8313	0.6968	0.9111
	FCGR + PseTNC	0.8439	0.8702	0.8178	0.6893	0.9111
	All features	0.8474	0.8909	0.8044	0.6980	0.9141
XGBoost	FCGR	0.8602	0.897	0.8239	0.7235	0.9237
	FCGR + DAC	0.8561	0.8627	0.8496	0.7130	0.9186
	FCGR + TAC	0.8865	0.9035	0.8696	0.7734	0.9394
	FCGR + DACC	0.8439	0.8667	0.8213	0.6894	0.9136
	FCGR + TACC	0.8406	0.8711	0.8104	0.6831	0.9046
	FCGR + PseDNC	0.8563	0.8931	0.82	0.7152	0.9208
	FCGR + PseTNC	0.8504	0.8712	0.8300	0.7029	0.9185
	All features	0.8511	0.8755	0.827	0.7039	0.9193
MLP	FCGR	0.8579	0.8839	0.8322	0.7172	0.9186
CNN	FCGR	0.8616	0.8746	0.8487	0.7239	0.9222

Best values are in bold

**Table 13:** Comparison of our predictors with other advanced models via 20-fold cross-validation for C. elegans

Method	Feature	ACC	Sn	Sp	MCC	AUC
LeNup [25]		**0.9188**	**0.9339**	**0.9041**	**0.8444**	**0.9663**
3LS [22]		0.8786	0.8654	0.8921	0.7576	0.9605
SVM	FCGR	0.8623	0.8946	0.8304	0.7268	0.9301
	FCGR + DAC	0.8578	0.8882	0.8278	0.7173	0.9274
	FCGR + TAC	0.8564	0.8804	0.8328	0.7143	0.9234
	FCGR + DACC	0.8483	0.8703	0.8266	0.6982	0.9170
	FCGR + TACC	0.8481	0.8726	0.824	0.6975	0.9201
	FCGR + PseDNC	0.8585	0.8948	0.8228	0.7198	0.9278
	FCGR + PseTNC	0.8551	0.8859	0.8248	0.7133	0.9285
	All features	0.8450	0.8359	0.8539	0.6900	0.9210
ELM	FCGR	0.8757	0.8940	0.8577	0.7525	0.9419
	FCGR + DAC	0.8715	0.8882	0.8551	0.7444	0.9371
	FCGR + TAC	0.8711	0.8878	0.8547	0.7433	0.9356
	FCGR + DACC	0.8715	0.8901	0.8532	0.7442	0.9374
	FCGR + TACC	0.8692	0.8948	0.8439	0.7400	0.9342
	FCGR + PseDNC	0.8678	0.8875	0.8486	0.7369	0.9348
	FCGR + PseTNC	0.8563	0.8851	0.8279	0.7144	0.9293
	All features	0.8614	0.8921	0.8312	0.7251	0.9299
XGBoost	FCGR	0.8520	0.8831	0.8213	0.7060	0.9207
	FCGR + DAC	0.8541	0.8855	0.8232	0.7108	0.9223
	FCGR + TAC	0.8537	0.8757	0.8321	0.709	0.9195
	FCGR + DACC	0.8465	0.8671	0.8255	0.694	0.9161
	FCGR + TACC	0.8471	0.8702	0.8244	0.6959	0.9188
	FCGR + PseDNC	0.8487	0.8781	0.8198	0.6996	0.9204
	FCGR + PseTNC	0.8501	0.8804	0.8202	0.7022	0.9224
	All features	0.8518	0.8851	0.8190	0.7059	0.9188
MLP	FCGR	0.8589	0.8864	0.8318	0.7206	0.9281
CNN	FCGR	0.8529	0.8778	0.8284	0.7076	0.9181

Best values are in bold

**Table 14:** Comparison of our predictors with other advanced models via 20-fold cross-validation for D. melanogaster

Method	Feature	ACC	Sn	Sp	MCC	AUC
LeNup [25]		**0.8847**	**0.8974**	**0.8713**	**0.7828**	**0.9401**
3LS [22]		0.8341	0.8407	0.8274	0.6682	0.9147
SVM	FCGR	0.8117	0.7841	0.8396	0.6251	0.8782
	FCGR + DAC	0.8094	0.7876	0.8316	0.6201	0.8783
	FCGR + TAC	0.8118	0.8073	0.8163	0.6252	0.8863
	FCGR + DACC	0.7866	0.7997	0.7733	0.5738	0.8384
	FCGR + TACC	0.7767	0.7128	0.8418	0.5593	0.8412
	FCGR + PseDNC	0.8095	0.7992	0.8199	0.6209	0.8843
	FCGR + PseTNC	0.8108	0.8034	0.8183	0.6234	0.8848
	All features	0.7602	0.7014	0.8200	0.5255	0.8059
ELM	FCGR	0.7912	0.7651	0.8204	0.5842	0.8601
	FCGR + DAC	0.7924	0.7752	0.8098	0.5862	0.8689
	FCGR + TAC	0.7932	0.7776	0.8091	0.5877	0.8619
	FCGR + DACC	0.7793	0.7710	0.7877	0.5599	0.8537
	FCGR + TACC	0.7697	0.7686	0.7709	0.5403	0.8456
	FCGR + PseDNC	0.7910	0.7659	0.8165	0.5837	0.8648
	FCGR + PseTNC	0.7691	0.7455	0.7930	0.5395	0.8433
	All features	0.7878	0.7859	0.7899	0.5763	0.8637
XGBoost	FCGR	0.8037	0.7821	0.8257	0.6088	0.8771
	FCGR + DAC	0.7891	0.7741	0.8042	0.5791	0.8648
	FCGR + TAC	0.7948	0.7762	0.8137	0.5910	0.8690
	FCGR + DACC	0.7814	0.7790	0.7839	0.5634	0.8540
	FCGR + TACC	0.7706	0.7648	0.7765	0.5417	0.8508
	FCGR + PseDNC	0.8010	0.7786	0.8239	0.6036	0.8728
	FCGR + PseTNC	0.8074	0.7958	0.8193	0.6165	0.8831
	All features	0.7979	0.7745	0.8218	0.5972	0.8739
MLP	FCGR	0.8127	0.8003	0.8253	0.6272	0.8893
CNN	FCGR	0.8116	0.8036	0.8198	0.6252	0.8854

Best values are in bold

LeNup has the best overall prediction effect. The accuracy of C. elegans is 0.9188, and the average accuracy of other species are also over 0.88. The prediction result of our method is relatively close to it on the H. sapiens dataset. For C. elegans, ELM with FCGR performs slightly worse than 3LS, ACC, $S_{p}$, MCC, AUC decreased by 0.29%, 3.44%, 0.51%, 1.86% respectively.

## Discussion

Firstly, the results in Table 1 and Fig. 1 clearly showed that the FCGR feature of the combined K value is better than the single K value, and the SVM output better prediction results. When training CNN and MLP models, we utilized multi-channel multiple K-value input images, and the prediction accuracy had been improved. All these indicated that FCGR feature combinations with different K values can better express sequence features, thereby improving models' prediction accuracy.

Secondly, we further integrated FCGR with other feature representations, and combined three types of machine learning algorithms to compare prediction results (Tables 2, 3, 4). Besides, we performed PCA dimensionality reduction processing on feature vectors to prevent high-dimensional features from causing overfitting (Tables 5, 6, 7). Although the overall prediction quality has improved after the PCA dimensionality reduction processing with the integrated feature, superior results are obtained for using FCGR feature representation. These also further illustrated the advantages of FCGR features representation.

Here we compared the results of the proposed method with other advanced algorithms. Slightly superior results are achieved with our algorithm on H. sapiens and S. cerevisiae datasets, but there are gaps in the other two datasets. On the one hand, it explains the feasibility of our method; on the other, our work has room for improvement.

## Conclusions

In this work, we used FCGR to represent the features of the DNA sequence and applied it to the nucleosome positioning. Our experiments have achieved positive results. Especially when multiple features are used in combination, the prediction quality can be improved. The advantage of this representation is that the time consumed in the process of constructing features is shortened, and the features are clear and intuitive. The quality of integrating features representation is also acceptable. Particularly after we use PCA for dimensionality reduction, the prediction quality of H. sapiens dataset has been improved. This demonstrates the feasibility of the method.

In this paper, we also tried a simple CNN model with FCGR image and got mediocre results. Since deep learning is now increasingly used in bioinformatics. In the further research of nucleosome positioning, we will try to build a more efficient deep learning prediction model to achieve prediction of DNA represented in the form of images, such as FCGR image.

## Methods

### Dataset descriptions

To compare the results of the predictors, the datasets of this work downloaded from two published papers [20, 21]. The first group of datasets involved H. sapiens, C. elegans and D. melanogaster from the paper by Guo et al. [21]. The length of each DNA sequence is 147 bp. The second dataset involved S. cerevisiae genome from the paper by Chen et al. [20]. The length of each DNA sequence is 150 bp. Both of the datasets contain two types of samples: nucleosome-forming sequences (positive data) and nucleosome-inhibiting sequences (negative data). And none of the sequences included has ≥ 80% pairwise sequence identity with any other. The details of the datasets are shown in Table 15.

### DNA sequence feature representation

Except for the above mentioned, common DNA sequence representation methods include basic kmer (Kmer) [34], reverse complementary kmer (RevKmer) [35], etc. based on deoxyribonucleic acid composition, and some are based on the correlation between nucleotide physical and chemical indicators, such as dinucleotide-based autocovariance (DAC), trinucleotide-based autocovariance (TAC) [29], etc. and pseudo k-tuple nucleotide composition (PseKNC) [21] based on pseudo deoxyribonucleic acid composition. These feature representation methods have specific calculation formulas and iterative functions, and some calculations are more complex and require a long time. This paper will mainly use a simple and intuitive feature representation.

Chaos game representation (CGR) is a graphical representation method of gene sequence based on chaos theory proposed by Jeffrey in 1990 [36]. The method is as follows: The four nucleotides {A, T, G, C} are located at the four vertices of the plane coordinate system, and the position of each nucleotide in the DNA sequence in the plane is $P_{i}$. According to formula (2) draw the coordinate point of each nucleotide:

2 $$P_{i} = 0.5 \cdot (P_{i - 1} + N_{i} ),\quad i = 1, \ldots { },L\;and\;P_{0} = (0.5,{ }0.5)$$

**Fig. 5:**
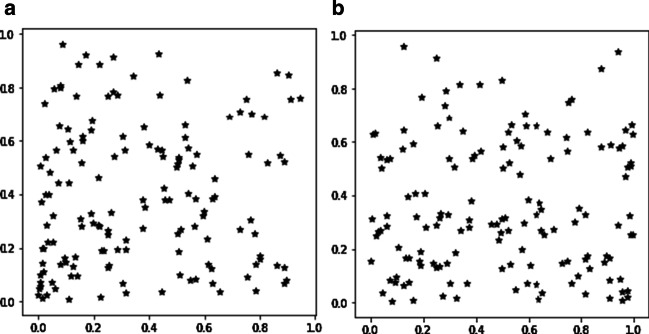
CGR of DNA sequences: **a** H. sapiens nucleosome-inhibiting sample and **b** H. sapiens nucleosome-forming sample

Among them, $P_{0}$ is the given starting point, L is the length of the DNA sequence, and $N_{i}$ represents the corresponding coordinate of the i-th nucleotide, where A = (0,0), T = (1,0), G = (1,1), C = (0,1). This method draws a corresponding image of a DNA sequence through the iterative function and makes the nucleotides in the sequence correspond to the points on the image one by one [36–40]. From Fig. 5, we can see the CGR graphical representation of the two types of sample sequences in the H. sapiens dataset.

Divide the CGR image into $2^{K} \times 2^{K}$ sub-blocks and calculate the number of points appearing on each sub-block, we can determine the frequency of K nucleotide combinations, and then convert the CGR image into a $2^{K} \times 2^{K} { }$ matrix, which is called frequency chaos game representation (FCGR) [39]. For example, we divided the CGR graph of Fig. 5a into a $2^{3} \times 2^{3}$ matrix and calculated the number of occurrences of the midpoint of each sub-block, and obtain the frequency matrix shown in Table 16.

**Table 15:** The quantity composition of the four species datasets

Species	N-f	N-i	Total
H. sapiens	2273	2300	4573
C. elegans	2567	2608	5175
D. melanogaster	2900	2850	5750
S. cerevisiae	1880	1740	3620

N-f indicates nucleosome-forming sequences (positive data) and N-i indicates nucleosome-inhibiting sequences (negative data)

**Table 16:** The frequency matrix of CGR image on H. sapiens nucleosome-inhibiting sample

1	3	1	1	0	0	0	0
3	1	4	1	1	0	2	3
3	2	0	1	1	2	2	0
4	4	3	4	7	0	2	2
5	1	0	4	2	3	0	0
3	7	2	2	5	1	2	0
8	4	5	0	4	0	4	1
12	2	2	1	3	2	2	2

**Fig. 6:**
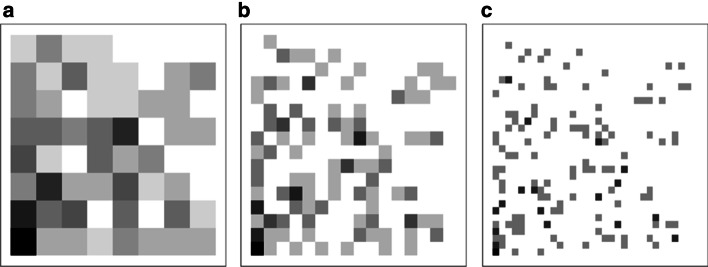
FCGR image of H. sapiens nucleosome-inhibiting sample with different K: **a** K = 3, **b** K = 4 and **c** K = 5

FCGR can be used not only as a numerical matrix, but also as a grayscale image. The original CGR image is divided into $4^{K}$ sub-blocks. The darker the sub-block, the more dots appear in the sub-blocks; the lighter sub-blocks, indicates that the number of dots in the color block is small, and the pixel value of the image is between 0 and 255 [39]. From Fig. 6, we can see the FCGR image of the sample sequence with K = 3, 4 and 5, respectively.

### Support vector machine

Support vector machine (SVM) is a commonly used two-class classification model. Compared with other classification algorithms, it has a good classification effect and strong generalization ability on small data sets. It can also handle nonlinear classification problems through nuclear techniques. Thus, support vector machines have also been widely used in the field of bioinformatics [19, 21, 23]. Its basic idea is to map the sample from the original low-dimensional space to a high-dimensional space, so that the sample can find a partitioning hyperplane with the largest interval in the feature space, and separate samples of different categories.

In this paper, we will use the python package (Scikit-learn 0.23), which can be downloaded from https://scikit-learn.org/stable/index.html. This package contains the SVM module, and the implementation is based on libsvm. We will train the SVM with the radial basis function (RBF) kernel, meanwhile two parameters will be considered: penalty parameter C and kernel coefficient Gamma. In the training process, we used the grid optimization method to determine the best values of the two parameters.

### Extreme learning machine

**Fig. 7:**
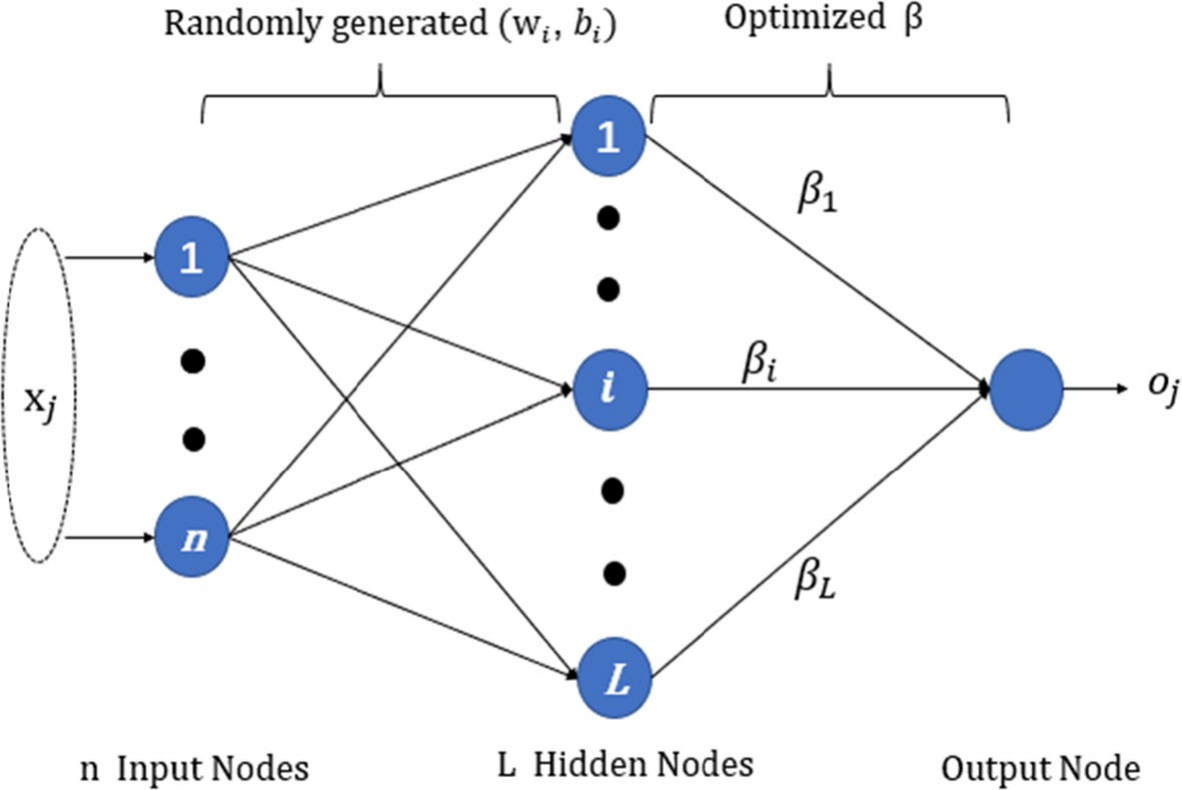
Basic architecture of ELM

Extreme learning machine (ELM) was proposed by Guang-Bin Huang. The algorithm is a new machine learning algorithm based on single hidden layer feedforward neural networks (SLFNs). Compared with traditional algorithms, ELM has a faster learning speed while maintaining learning accuracy. The core idea is to randomly select the input layer weight and hidden layer bias of the network, and get the corresponding hidden node output [41]. The network structure of ELM model is shown in Fig. 7.

The experiment reference used David Lambert's Python version of ELM resources, which can be downloaded from the ELM web portal (https://www.ntu.edu.sg/home/egbhuang/). The code can be found on https://github.com/dclambert/Python-ELM.

### Extreme gradient boosting

Extreme gradient boosting (XGBoost) is an open source machine learning project developed by Tianqi Chen et al. [42]. It is one of the boosting algorithms, which has the characteristics of high efficiency, flexibility, high accuracy, and strong portability. It is applied in the field of biomedicine [43].

The idea of XGBoost algorithm is to continuously add trees and perform feature splitting to complete the construction of a tree. In the whole process, each addition of a tree is learning a new function to fit the residual of the previous prediction. When the training is completed, K trees will be obtained. If we want to predict the score of a sample, according to the features of this sample, each tree will fall to a corresponding leaf node, and each leaf node corresponds to a score. Finally, we only need to add up the scores corresponding to each tree to get the predicted value of the sample.

In this experiment, we used the python package (xgboost 1.2.0), which can be downloaded from https://github.com/dmlc/xgboost.

### Multilayer perceptron

Multilayer perceptron (MLP) is also called deep neural networks (DNNs) [44]. MLP is based on the extension of perception. Multiple hidden layers are introduced between the input layer and the output layer, and the neurons between the layers are fully connected. So, both the hidden layer and the output layer in MLP are fully connected layers.

For the MLP, we used the AI Studio (https://aistudio.baidu.com/aistudio/index) experimental platform and PaddlePaddle (https://www.paddlepaddle.org.cn/) deep learning framework provided by Baidu (https://www.baidu.com/) to implement the experimental model with python (https://www.python.org/). MLP has three hidden layers with Relu activation function [45], each layer contains 50 neurons, the output layer uses a softmax activation function. Besides, MLP is trained by 5 epchos, with Adamax optimizer a learning rate of 0.001. Adamax algorithm is a variant of Adam algorithm based on infinite norm, which makes the algorithm of learning rate update more stable and simple [46]. We use cross entropy as our loss function.

### Convolutional neural network

Convolutional Neural Network (CNN) is a representative algorithm of deep learning. It has demonstrated extraordinary advantages in the field of computer vision and has also been widely used in bioinformatics [47, 48]. Convolutional neural networks can automatically extract features from input data. Compared with fully connected neural networks, it can simplify model complexity and effectively reduce model parameters [49]. Convolutional neural networks are applied to the general framework of image mode, mainly composed of convolutional layers, activation function, pooling layers and fully connected layers [49, 50].

**Fig. 8:**
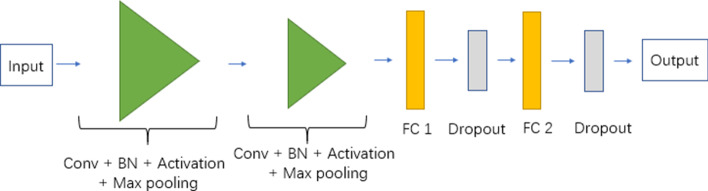
The architecture of our CNN model

Owing to the limitation of the sample data volume, during the training process, we need to prevent the over-fitting problem faced by CNN, so we add a batch normalization (BN) layer [51] after the convolutional layer and add a dropout layer [52] after the fully connected layer. In our network, the convolutional layer uses a 3 × 3 convolution kernel, the number of filters in the first layer is 64, and the second is 32. The pooling layer use the maximum pooling of 2 × 2, with stride = 2. The first fully connected layer neurons' number is 100, and the second is 50. Then, the dropout probability of the subsequent dropout layer is 0.5. Except the softmax activation function used in the output layer, the activation function in the other layers is Relu. CNN is training by 20 epchos, with Adamax optimizer a learning rate of 0.001. The loss function is cross entropy. Like MLP, we also used the AI Studio experimental platform and PaddlePaddle deep learning framework provided by Baidu to implement the experimental model in python. The specific network structure is shown in Fig. 8.

## Data Availability

The datasets of this work can be downloaded from two published papers [20, 21]. The python source code used in this work are freely available at https://github.com/lliqi-echo/Comparative-analysis-and-prediction-of-nucleosome-positioning.

## Abbreviations

ACC: Accuracy
AUC: Area under curve
CGR: Chaos game representation
CNN: Convolutional neural networks
DAC: Dinucleotide-based autocovariance
DACC: Dinucleotide-based auto-cross covariance
ELM: Extreme learning machine
FCGR: Frequency chaos game representation
MCC: Mathew's correlation coefficient
MLP: Multilayer perceptron
PCA: Principal component analysis
PC-PseDNC: Parallel correlation pseudo dinucleotide composition
PC-PseTNC: Parallel correlation pseudo trinucleotide composition
$${\text{S}}_{{\text{n}}}$$: Sensitivity
$${\text{S}}_{{\text{p}}}$$: Specificity
SVM: Support vector machine
TAC: Trinucleotide-based autocovariance
TACC: Trinucleotide-based auto-cross covariance
XGBoost: Extreme gradient boosting
